# Rapid Yeast Cell Viability Analysis by Using a Portable Microscope Based on the Fiber Optic Array and Simple Image Processing

**DOI:** 10.3390/s20072092

**Published:** 2020-04-08

**Authors:** Weiming Wang, Hang Liu, Yan Yu, Fengyu Cong, Jun Yu

**Affiliations:** 1 Faculty of Electronic Information and Electrical Engineering, Dalian University of Technology, No. 2 Linggong Road, Ganjingzi District, Dalian 116024, China; wwm_warmly@mail.dlut.edu.cn (W.W.); liuhang@dlut.edu.cn (H.L.); 17753853799@163.com (Y.Y.); congfy18@163.com (F.C.); 2 Key Laboratory of Integrated Circuit and Biomedical Electronic System of Liaoning Province, Dalian University of Technology, Dalian 116023, China

**Keywords:** FOA, lens, yeast activity, yeast concentration, portable microscope

## Abstract

A fiber optic array (FOA) can be used as an alternative or a supplement to the lens in a microscope due to its large magnification, high coupling efficiency and extremely low distortion. Based on our previous research, this paper first demonstrated the resolution and field-of-view (FOV) of the microscope based on the FOA. To further validate the FOA microscope’s imaging capability, yeast activity and concentration were investigated by simple image processing. The results showed that the percentages of live and dead yeast cells correctly identified were 92.1% and 84.8%, except for the clusters, which agreed well with the manual counting methods. Then, the performances of the portable microscopes based on the FOA and lens were compared and the factors that affect the FOA microscope imaging performance were analyzed.

## 1. Introduction

To address the demand for diagnosing parasites in remote areas and telemedicine, portable microscopes based on lenses have achieved rapid deployment due to their simple principles and operation [1,2,3,4,5,6,7]. However, due to the inherent characteristics of the objective lens, this microscope has limitations which include size (microscope barrel length [8]), large optical distortions or aberrations and tedious focusing. Although the distortion can be partly overcome by an achromatic objective lens, the demands for light-weight and cheap portable microscopes have limited its use. Furthermore, the current mobile microscopes are still unable to run complex algorithms, so do not meet the real-time requirements in many fields [9,10]. Therefore, a portable microscope with high resolution, a small volume and a real-time display is in high demand. As an alternative to the lens, a fiber optic array (FOA) can be used as an amplifier component due to its distortionless imaging, high magnification and high coupling efficiency, which provides a new idea for portable microscopes.

**Figure 1:**
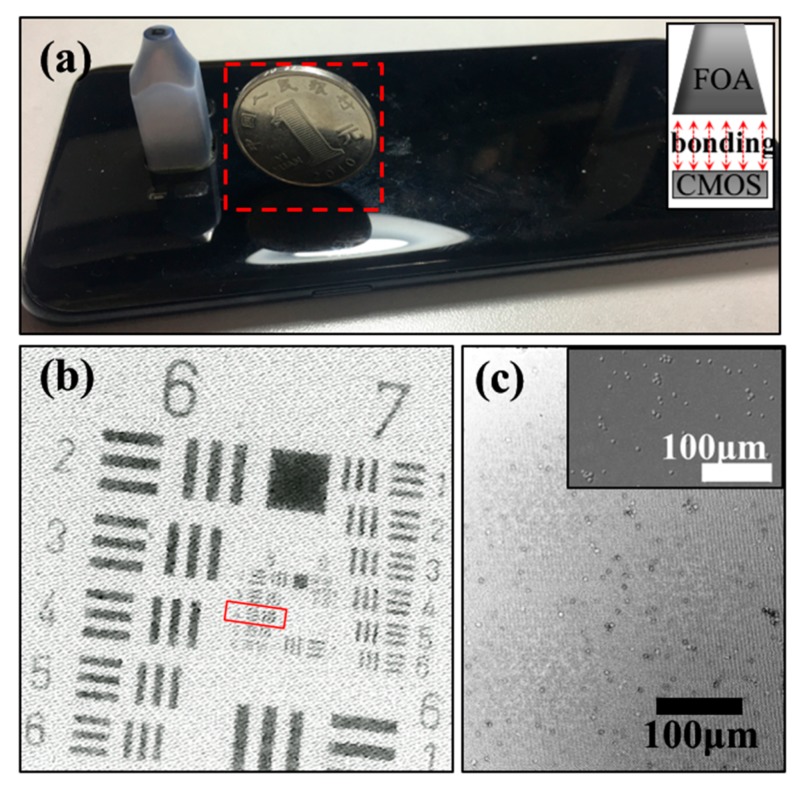
The FOA microscope and its imaging performance. (**a**) Photographs of the FOA microscope and schematic diagram (inset). (**b**) The related resolution and yeast cell test, while the inset is the image captured by using the commercial digital microscope. (**c**) The yeast cell detection and the corresponding commercial microscope (inset figure, Keyence).

**Figure 2:**
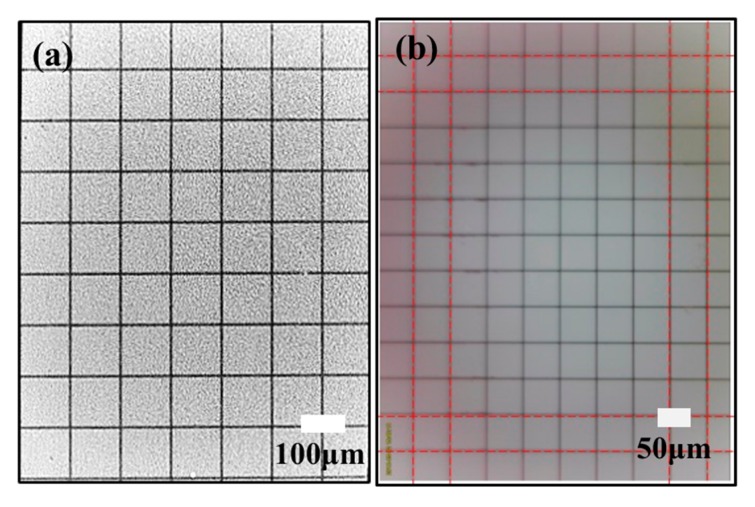
The FOV quantification of the images captured by using the FOA (**a**) and lens-based (10×) (**b**) microscope.

The FOA is constructed by a bundle of tapered optical fibers, and can be used to realize light transmission with the efficiency up to 70% as a light-coupled device [11]. It was previously used to effectively couple light into the Complementary Metal Oxide Semiconductor (CMOS) or charge coupled device (CCD) sensor in the low-light night vision of the military [12,13]. In biomedical fields, the FOA has been widely applied in medical imaging and medical X-ray inspection [14]. As an alternative to the lens in the microscope, FOA can be used to magnify the image of the specimen that is placed on the small end of FOA, and then project it to the CMOS sensor [15]. This solution reduces the device’s volume and improves the light transmission efficiency [16]. Our research group has addressed this issue by mounting the FOA (Honsun, China, magnification = 6×, with the diameters of 1.5 μm and 9 μm at the small end and large end of the FOA, respectively) on a smartphone camera (S7, SAMSUNG, with CMOS pixel size of 1.4 μm) to develop a portable FOA microscope (Figure 1a) [17]. The full-pitch (line-pair) resolution of FOA microscope is 3 μm (Figure 1b), which is equivalent with the lens microscope (10×). Meanwhile, the FOV quantified by the grid distortion target (Thorlabs, R1L3S3P) is 950 × 700 μm (Figure 2a) which is 2.24 times larger than the latter (625 × 475 μm, Figure 2b), while the volume is greatly reduced. The distortion is invisible to the naked eye. The yeast cell images (Angel Yeast co. ltd., China) obtained by using the FOA microscope are shown in Figure 1c. The inset captured by the commercial microscope (Keyence, VH-Z100RRZ100) is shown in the figure for reference.

## 2. Yeast Activity and Counting Detection by the FOA Microscope

Yeast is widely used in the alcohol, baking and biofuel industries. Fast and real-time monitoring the activity of yeast can effectively avoid economic loss caused by fermentation failure [18,19,20,21]. The traditional yeast monitoring method is time-consuming, and done by employing of a hemocytometer or flow cytometer [22]. By using a support vector machine (SVM), auto-focusing processes and holographic microscopy, the Ozcan group first took 5–10 mins to automatically classify the cell viability and the results agree very well with the traditional gold-standard method. However, the above professional operation has limited its popularization [23]. Our FOA microscope offered a portable automatic yeast monitoring platform (Figure 1a) to obtain the concentration and activity of dead cells and live cells. Meanwhile, professional operation is not required for our FOA microscope, including the machine learning algorithm and complex operations. Preliminary results can be obtained by the traditional image preprocessing method, including adaptive histogram equalization (AHE) and image scan convolution [24].

### 2.1. Sample Preparation

The active dry yeast (Angel yeast, China) was rehydrated in distilled water and equally divided into two parts for the dead and live yeast cells, respectively. The dead yeast cell was obtained by heat-killing. Then, 1:1 volume of 0.1% Methylene Blue was added to the solution to stain the dead cells. Then the solution was mixed with the rehydrated live yeast cells. A drop of the solution was dripped onto the slide by the microsyringe. Lastly, the liquid drop was placed on the FOA small end of the microscope and the cover slip was overlain on the top to reduce the volatilization.

### 2.2. AHE Processing

**Figure 3:**
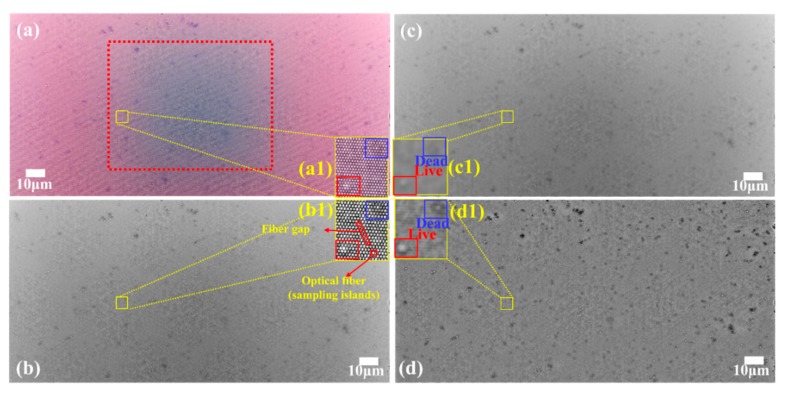
Yeast activity and concentration detection. (**a**) The original yeast image obtained by the FOA microscope; (**b**) the corresponding grayscale image. (**c**) The grayscale image processed by average filtering (radius = 5); (**d**) the grayscale image after AHE processing. The yellow label indicates the optical fiber gaps.

When used for yeast cell detection, two problems have to be solved by the FOA microscope: the artifacts of fiber gaps (Figure 3(b1)) and the uneven illumination artifacts. Regarding Figure 3(a1), the FOA imaging was similar to the CMOS contact imaging, whose pixels arranged regularly. The difference was that larger lighttight gaps existed among the optical fibers (Figure 3(a1,b1)), which significantly interfered with the cell contour recognition. To address this issue, preprocessing, including enhancing the cell profile and weakening the fiber gaps, was essential. In the experiment, traditional image smoothing, including mean and median filtering, proved to be invalid, since the contours of yeast cell and the fiber core gaps (Figure 3(c1)) were both filtered as the noise. Meanwhile, in the frequency domain, cell contours were also identified as high frequency noise, which was not effective for the recognition. Therefore, the traditional image denoising methods were not applicable for the FOA microscope.

In addition, limited by the size and cost of the portable microscope, a commercial, cheap broad-spectrum LED was used to provide illumination. This may be not as mature as the commercial benchtop microscope and caused uneven illumination. Meanwhile, the big end of FOA was cut to match the CMOS’s photosensitive area, which led to increased incident light. Therefore, the image showed an uneven illumination distribution in the Figure 3a. The middle region of the photomicrograph was gray, while the surrounding area was white. The corresponding grayscale image (Figure 3(b1)) demonstrated that uneven illumination seriously affected cell recognition performance.

To solve the above two problems in the FOA microscope, the local adaptive histogram equalization (AHE) has been applied to the gray image (Figure 3b). The result proved that uneven illumination was greatly improved in the Figure 3d, while cell contour was enhanced simultaneously. Several histograms were computed and each histogram corresponded to a distinct section of the image. Then, they were used to redistribute the lightness values of the image [25]. The cell profile and contrast (Figure 3(d1)) was enhanced and illumination unevenness was significantly improved compared with the gray image (Figure 3(b1)). “Sampling islands” have been removed.

**Figure 4:**
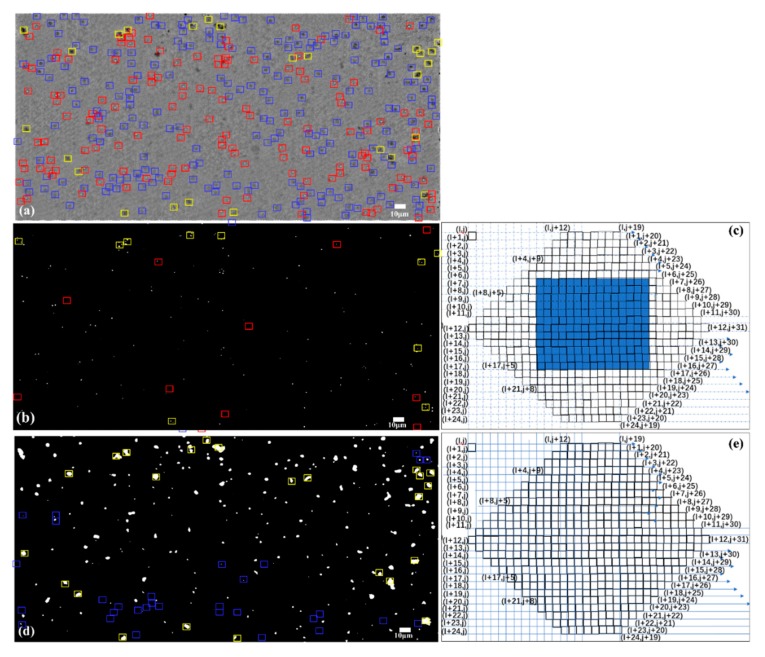
The yeast cell concentration quantified by the scan window filter. (**a**) The original grayscale image using AHE processing with the manually labeled live (red) and dead (blue) cells, respectively. (**b**), (**d**) The filtered live and dead cells shown by using the live-cell-convolution and the dead-cell-convolution windows. (**c**), (**e**) The scan windows for live and dead detection, respectively. The red and blue label in (**b**,**d**) indicate false detections, while the yellow labels are the cell clusters.

The corresponding scan windows are designed for the classifications of the live cells (Figure 4c) and dead cells (Figure 4e), respectively. The detailed descriptions are as follows:

For the live cell screening in Figure 4c, the hexagons are designed for the convolution window to filter out the live cells based on cell outlines. We found that when comparing the pixel values of lots of living cells, the middle area has a brighter white area with a higher gray value and an opposite gray value on the edge in Figure 3(d1). The contrast of gray value is larger than that of the dead cell and the background. When performing the scanning operation, the difference operation between the sum of the edge gray values and the sum of the inner squares values is performed and saved in the matrix. The point with the largest difference is finally filtered and retained in the original image. In the figure, the first pixel in the upper left corner is (i, j), which represents the coordinate position of the pixel in the convolution window, and the coordinates are incremented row-by-row and column-by- column. This convolution window is used to scan the microscopic image and to keep the location of the live cells.

For the dead cell screening in the Figure 4e, after observing the pixel value characteristics of dead cells, we found that the sum of the gray values in the dead cell region was lower than that of the background area and the living cell area in the Figure 3(d1). Therefore, when performing a convolution scan, the sum of the hexagonal areas is calculated and the sum is stored in the matrix. Finally, the point with the lowest pixel value of dead cell is selected.

The ordinary histogram equalization algorithm uses the same histogram transformation for the pixels of the entire image. This algorithm works well for those images with a relatively uniform distribution of pixel value. However, if portions in the image are significantly darker or brighter than other areas of the image, the contrast for these portions will not be effectively enhanced. In the AHE algorithm, each pixel is equalized by a histogram of pixels within a rectangular range around it. The transformation function is proportional to the cumulative histogram function (CDF) around the pixel.

**Figure 5:**
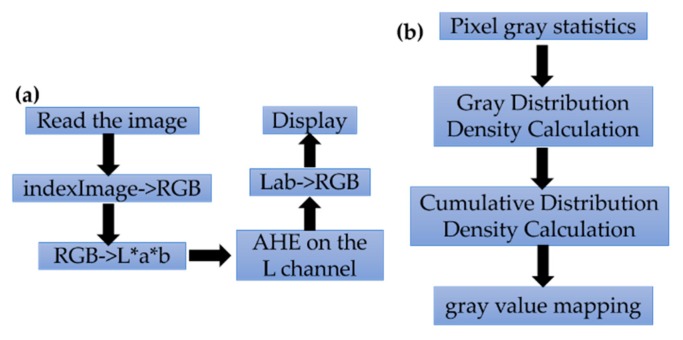
The overall optimization procedure: (**a**) the overall AHE optimization; (**b**) the detail of the AHE process.

The overall optimization procedure is described as follows in the Figure 5a: (1) Read the indexed color image into the workspace; (2) convert the indexed image into a truecolor (RGB) image, and then convert the RGB image into the L*a*b* color space; (3) scale values to the range expected by the adapthisteq function, [0 1]; (4) perform CLAHE on the L channel, and scale the result to get back to the range used by the L*a*b* color space; (5) convert the resulting image back into the RGB color space.

In detail, for the AHE process, it uses the histogram matching method to process smaller areas (called small blocks) in the image one by one. Bilinear interpolation is then used to combine adjacent patches to eliminate the boundaries introduced by the entrance. 

***The Algorithm’s Principle***

The template w is moving line-by-line on the image *A*, and the center pixel value $c(x_{0},y_{0})$ of the template w corresponds to the pixel value $f(x_{0},y_{0})$ on the image; the histogram equalization change of the template w area is calculated by using the following:

(1) g(x,y)=T(f(x,y))

By using the above expression, the gray histogram of the area is changed from a certain gray interval set to a uniform distribution over the entire gray range (Figure 5b). Then the equalized pixel value of the template center pixel value $c(x_{0},y_{0})$ is calculated by using the following:

(2) $$g(x_{0},y_{0})=T(f(x_{0},y_{0}))$$

$f(x_{0},y_{0})$ is replaced by $g(x_{0},y_{0})$. At last, the optimized image of the whole image is obtained by line-by-line calculation.

The function listed as follows in the OpenCV library is used to perform the AHE process and specifies the additional parameter/value pairs. The OpenCV library was then ported to the android app that installed on the smartphone.

*g* = *adapthisteq* (*image, param1, val1, param2, val2, param2*)

For the function, all the parameters are listed as follows: “*NumTiles*,” “*ClipLimit*,” “*NBins*,” “*Range*,” “*Distribution*” and “*Alpha*.” For our FOA microscope image, “*NumTiles*” and “*ClipLimit*” are the required parameters after experimental test. The parameter/value pairs of param1-val1 are set as “*NumTiles*” [25, 25]; that is a two-element vector consisting of a positive integer, with the number of small pieces specified by a row × column of 25 × 25 of the vector for the above blocks. This value determines the small block area used for each appropriate histogram equalization. The param2-val2 are set as “*ClipLimit*”: 0.1; that is used to specify the limits of contrast enhancement ranging from 0.01 to 1. Higher values produce stronger contrast. The value of 0.1 is proven to be effective.

### 2.3. Scan Window for Cell Detection

By using the AHE processing, the edges of optical fiber core almost disappeared while the yeast cell contour was deepened in Figure 3(c1). To obtain the number of live and dead yeast cells, two steps including the cell location determinations and the cell counting were proposed. Firstly, scanning windows matching the cell shape were designed for the live and dead yeast cells in Figure 4c,e respectively, and the locations that match the cell characteristics were reserved in the Figure 4b,d. Then, automated counting of the live and dead cells was performed by the software (ImageJ, National Institutes of Health). The bright area up to two pixels (with no upper size limit) can be recognized as a cell by using the “Analyze Particles” function.

Manual labeling and counting were performed in the Figure 4a for the comparison, and the numbers of live and dead yeast cells were 139 and 214 except for the cell clusters, respectively. Meanwhile, the corresponding counts by the FOA microscope were 129 and 183, respectively. Those results demonstrated that the percentages of unstained live cells and stained dead cells identified were 92.8% and 85%, respectively. Five-fold cross-validation was further performed on 100 images for activity and concentration detection. The results for live and dead yeast cells identified accounted for the percentages of 92.1% and 84.8%. The computational process takes about 5 s by using the ImageJ software on a laptop with an Intel Core i5 CPU at 2.40 GHz with 8 GB RAM. In comparison with the UCLA group (96.3% and 96.9% for the unstained and stained cells identified) [24], this detection accuracy achieved by the FOA microscope may be lower than the latter. However, the AHE and scan window operation can be run on a mobile phone without machine learning processing. An almost real-time presentation of results can be obtained which is suitable for the ordinary alcohol beverage and baking industry. For a larger number of solution detections, several images can be stitched to form a large FOV.

## 3. The Factors That Affect the FOA Microscope’s Performance

The resolution of a FOA-based microscope is directly related to the transmittance and resolving power of the FOA, resolving power of the CCD [26] and the coupling efficiency between the FOA and CMOS [17]. We demonstrated that it is easier to satisfy the total reflection condition when the incident light transmits into the small end than the end. The related factors that contribute to improving the resolution are as follows.

### 3.1. The Design Parameters of the FOA

**Figure 6:**
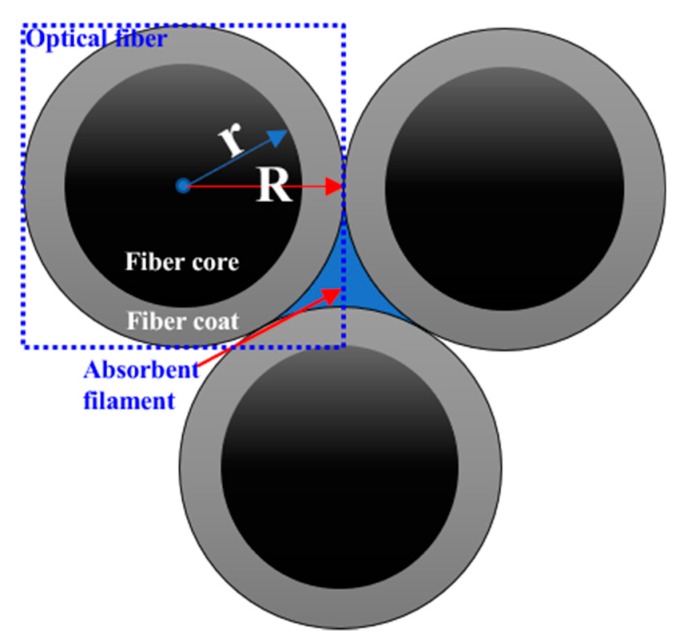
Optical fiber arrangement.

**Figure 7:**
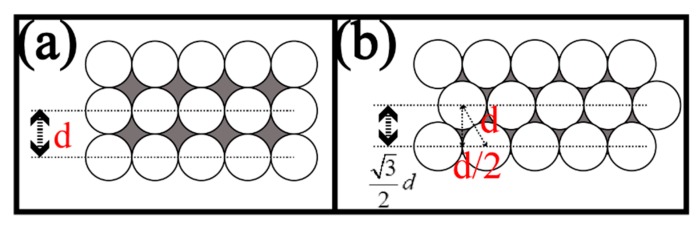
The square (**a**) and hexagonal (**b**) arrangements of fiber cores in the FOA, where d is the radius of the fiber core.

When the fiber cores are arranged regularly and the optical insulation is good, the resolution of the FOA is mainly affected by two factors: the arrangement between adjacent fibers and the core center distance. FOA is used as the first-stage imaging amplifier device of the coupled FOA microscope. Therefore, its resolution has a huge impact on the overall resolution of the microscope. The resolution of the FOA mainly depends on the center distance between the fiber cores, the arrangement, the scanning method and the fiber diameter. A smaller core diameter and closer fiber arrangement will provide higher theoretical resolution. However, the crosstalk among the cores and the total reflection in the optical transmission must be deliberated to reduce unnecessary transmission loss. Therefore, the design of the FOA needs to satisfy the ratio of cladding to core area of 65%/35%, as shown in Figure 6. That is, the core radius is 0.61 μm and the cladding thickness is 0.11 μm. In order to achieve a higher resolution, the core hexagonal arrangement shown in Figure 7 was employed. Its effective light transmission area was $\frac{\pi }{3.464}{\left(\frac{d}{D}\right)}^{2}$ (where d and D are the core radius and optical fiber radius, respectively), which can reach up to 60% effective transmission light area. The FOA can reach to a theoretical resolution of 0.95 μm according to the formula $1/\sqrt{3}d$. This arrangement is 1.15 times higher than the resolution obtained by the quadrangular arrangement (1/2d), which should help to overcome the diffraction limit caused by the large size of the CMOS pixel.

### 3.2. Tapered Fiber Made of Weakly Conducting Fibers Helps to Reduce the Distortion

Small transmission loss and signal distortion mean higher coupling quality and higher resolution [17]. When the fiber has core and cladding with a low refractive index difference, it can transmit a modulus with a small propagation angle. Meanwhile, the degree of evanescent-field into the coating is greater than that of the fiber with a large refractive index difference between the core and the coating. Normalized frequency formula for tapered fiber can be given by Equation (3) [27]:

(3) $$V_{taper}=\frac{2\pi r}{\lambda }\sqrt{n_{cl}^{2}-n_{ext}^{2}}$$

where r is the radius of the fiber core; λ is the incident wavelength; and $n_{cl}$ and $n_{ext}$ are the refractive index of the core and cladding. For our microscope, r = 0.6, λ = 550 nm, $n_{cl}$ = 1.52 and $n_{ext}$ = 1.5. The fiber parameter $V_{taper}$ is 0.41. This value is less than 2.405. Therefore, the core of the optical fiber can only transmit the fundamental mode $HE_{l1}$ [27]. The signal distortion of single-mode fiber is mainly caused by material dispersion, and the influence of multi-mode dispersion is small. Therefore, when designed as a single-mode fiber, it helps to reduce optical distortion when compared to a multi-mode fiber.

Meanwhile, as the radius of the tapered fiber gradually increases along the direction of the fiber axis, the normalized frequency also increases accordingly; that is, the number of fiber modes gradually increases. The formula proves that it is not easy to cause transmission light leakage, and the energy is more concentrated in the core when the incident light comes from the small end to the large end.

### 3.3. Effects of Crosstalk between Fibers, and Core and Cladding Thicknesses on Microscope Resolution

When the refractive index between the core and cladding is properly selected, the light transmitted into the fiber core will penetrate into the cladding with a small depth (about a few wavelengths), so it is essential to make a larger-thickness cladding to prevent the leakage of light.

For the optical fiber, the leakage of light will cause energy loss, leading to the loss of the information carried by the light, and then affect the signal-to-noise ratio and the optical signal, which will reduce the resolution. When light is transmitted into the small end of the fiber, the propagation path is analyzed as follows:

**(a)** When the light is transmitted into small end, the incident angle will gradually increase as the number of reflections increases (Equation (4)) [26]. For each reflection of light in the core, the transmission mode changes from high-order mode to low-order mode (Equation (4)). The incident angle $\varphi_{n}$ at the interface between the core and the cladding will be larger than the critical angle, which will not break reflection conditions. The light feature angle of corresponding mode decreases as the number of reflections increases. Moreover, it can be seen from the Equation (5) that the higher cone angle of the FOA will lead to faster changing incident angles, faster propagation mode changes from the higher-order mode to the lower-order mode and the lesser energy losses. This is huge advantage of incident light transmitted from the small end to the large end.

(4) $$\varphi_{n}=\frac{\pi }{2}-\frac{n_{0}}{n_{1}}\arcsin\theta_{s}+(2n-1)\frac{\delta }{2}$$

(5) $$\theta_{n}=\frac{n_{0}}{n_{1}}\arcsin\theta_{s}-(n-1)\alpha$$

where δ is the taper fiber cone angle, and n is number of reflections. $n_{0}$ and $n_{1}$ are the refractive indexes of the fiber core and cladding. α is the cone angle.

**(b)** When the light transmits from the small end, it also follows the Equation (6) for effective penetration depth of evanescent waves: The incident angle will gradually increase, so the penetration depth will decrease, which means that the less evanescent waves leak out, and the evanescent waves carry detail information in the near-field, which will cause an increase in the effective resolution of the fiber. That is, light is more difficult to penetrate through the core.

(6) $$d_{p}=\frac{\lambda }{2\pi }\frac{1}{\sqrt{n_{co}^{2}\sin^{2}\theta -n_{cl}^{2}}}$$

where $d_{p}$ is the effective penetration depth of the evanescent wave; $n_{co}$ and $n_{cl}$ are the refractive indexes of the cladding and the core; and θ is the angle of incidence light. λ is the wavelength of the incident light.

Therefore, the evanescent wave carrying the precise information of the specimen is not easily leaked out of the cladding, as the incident light transmitted into the small end of FOA. The influencing factors are independent of the thickness of the cladding.

## 4. Conclusions

Based on the previous research, this paper demonstrated the high resolution and low distortion of the FOA microscope. Meanwhile, yeast activity and concentration were detected by the FOA microscope with accuracies of 92.1% and 84.8% for identifying live and dead yeast cells, except for the cell clusters, which agreed well with the manual counting. Then, the performances of the portable microscopes based on the FOA and lens were compared and the factors that affect the FOA microscope imaging performance were analyzed.
